# Supplementary material for: Small protein modules dictate prophage fates during polylysogeny
Source: Nature. 2023 Jul 26;620(7974):625–33. doi: 10.1038/s41586-023-06376-y (PMC10432266; doi:10.1038/s41586-023-06376-y)
Supplement: Supplementary file 1 — Uncropped gels from representative and replicate experiments collected in this study. [file 41586_2023_6376_MOESM1_ESM.pdf]

nature portfolio

https://doi.org/10.1038/s41586-023-06376-y

Supplementary information

Small protein modules dictate prophage fates during polylysogeny

In the format provided by the authors and unedited

Supplementary Figure 1

1353_1

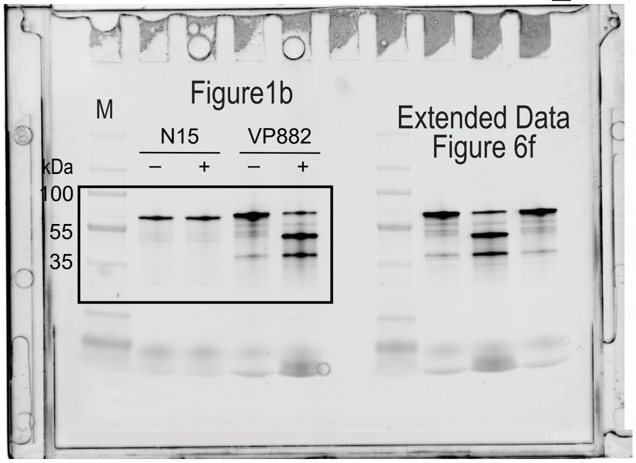

1123

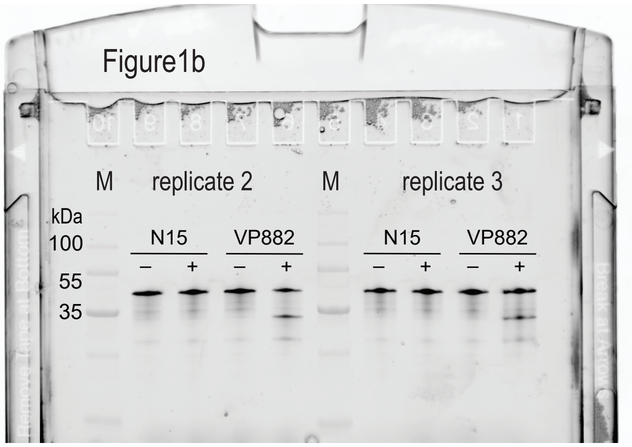

1351

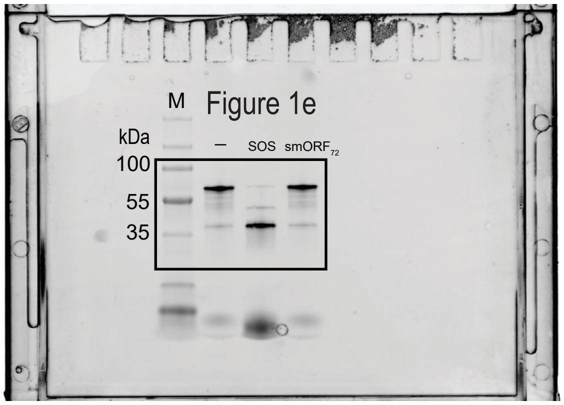

1348

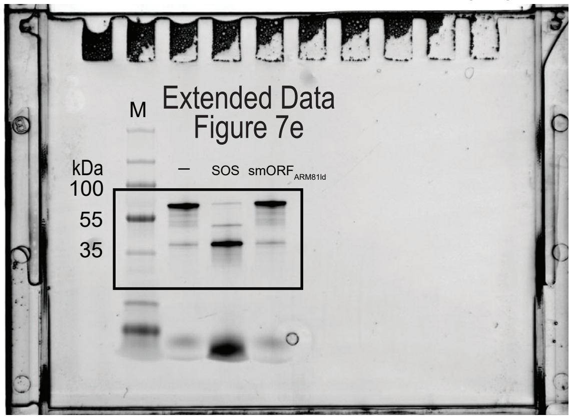

1349

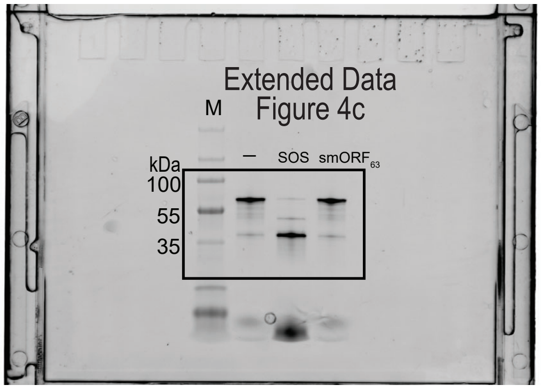

1353_2

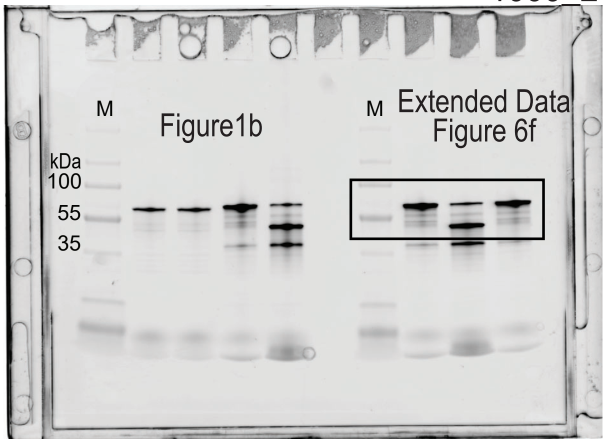

Figure 1b and 1e, and Extended Data Figure 4c, 6f, 7e replicates

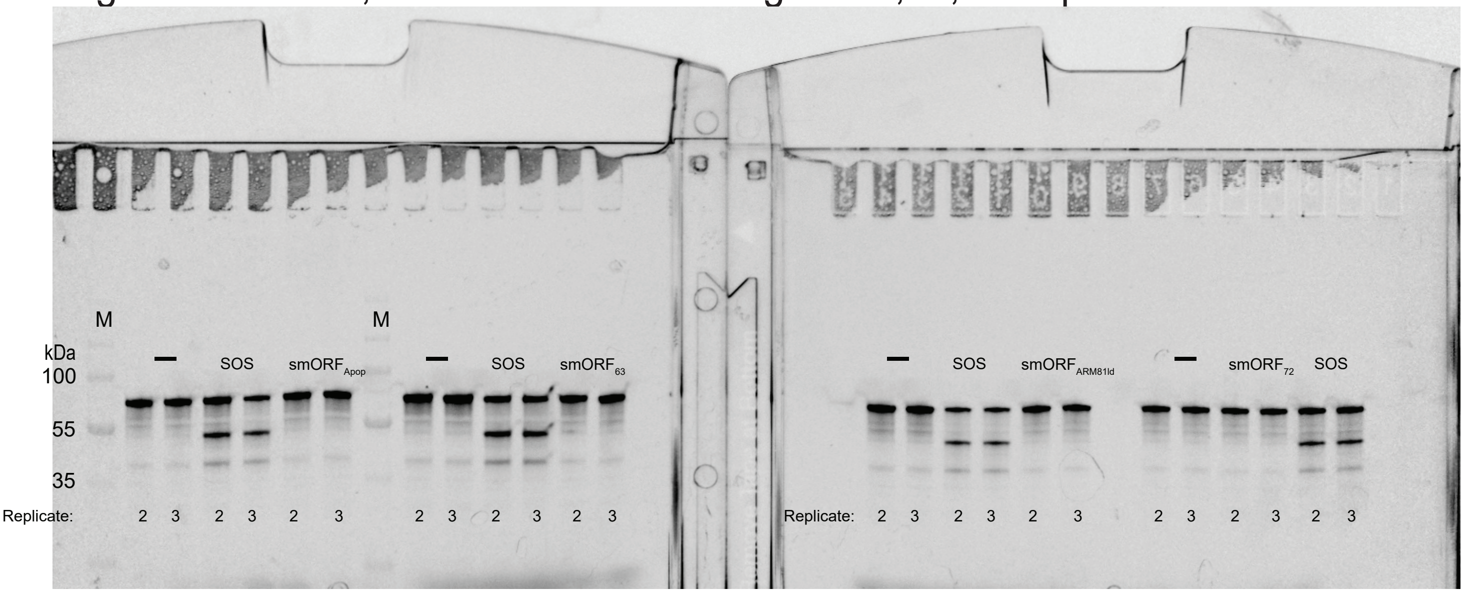

Extended Data Figure 1 and 4

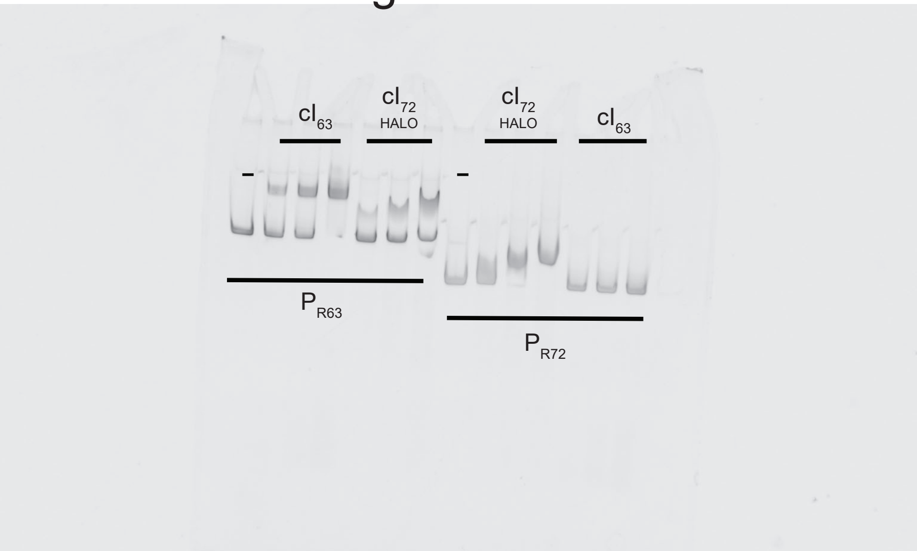

replicate 1

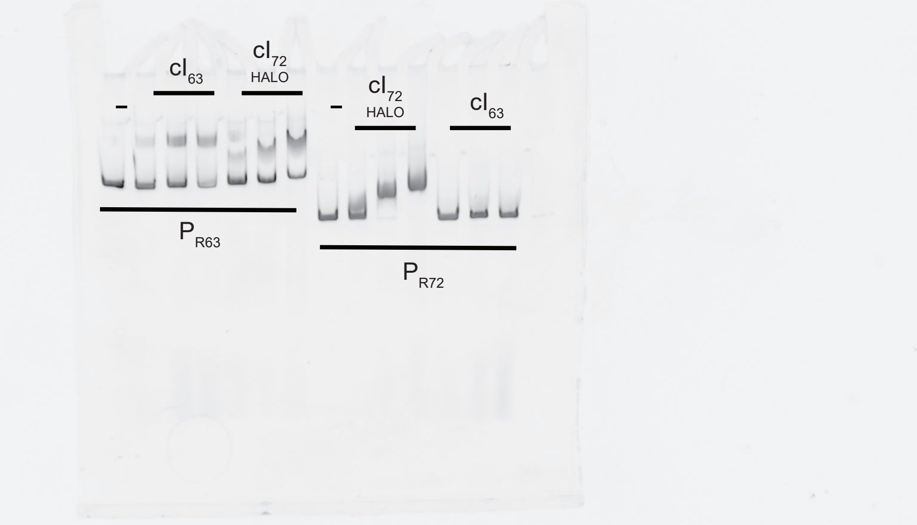

replicate 2

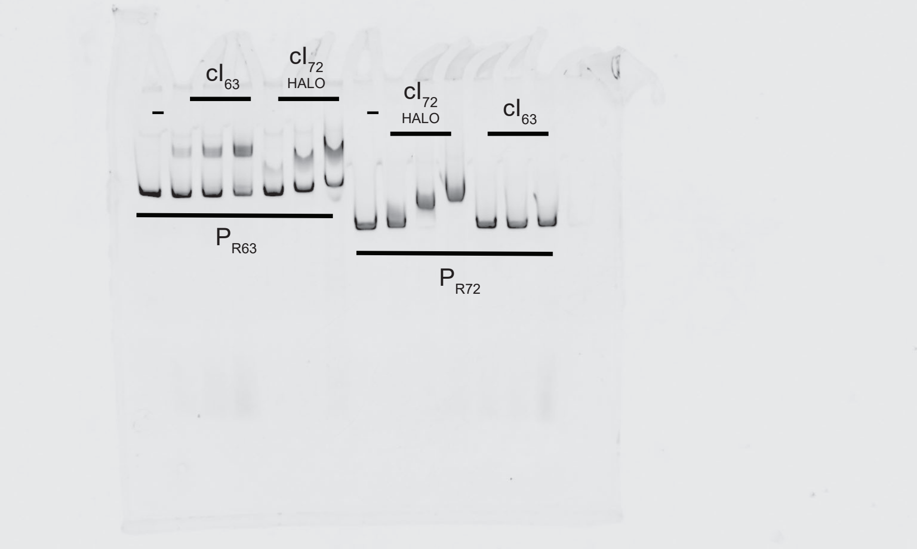

replicate 3

Extended Data Figure 6 and 7

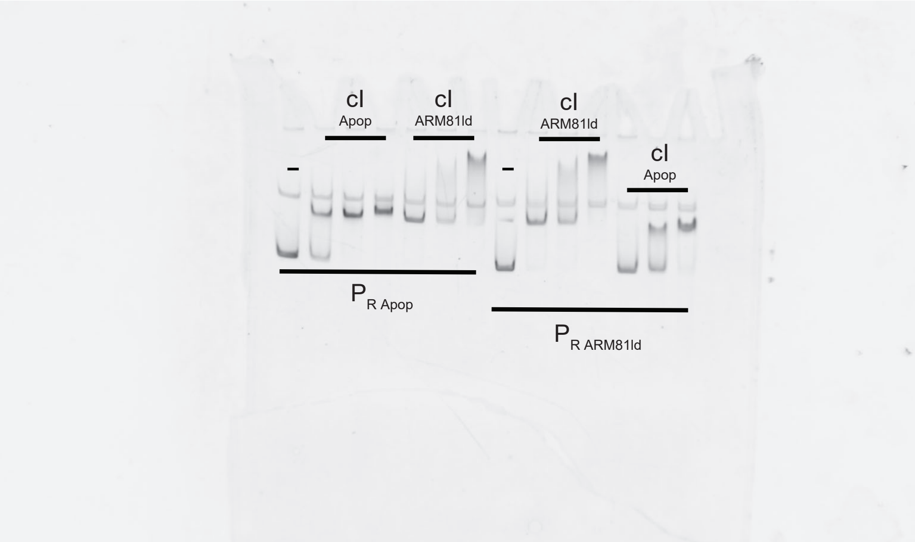

replicate 1

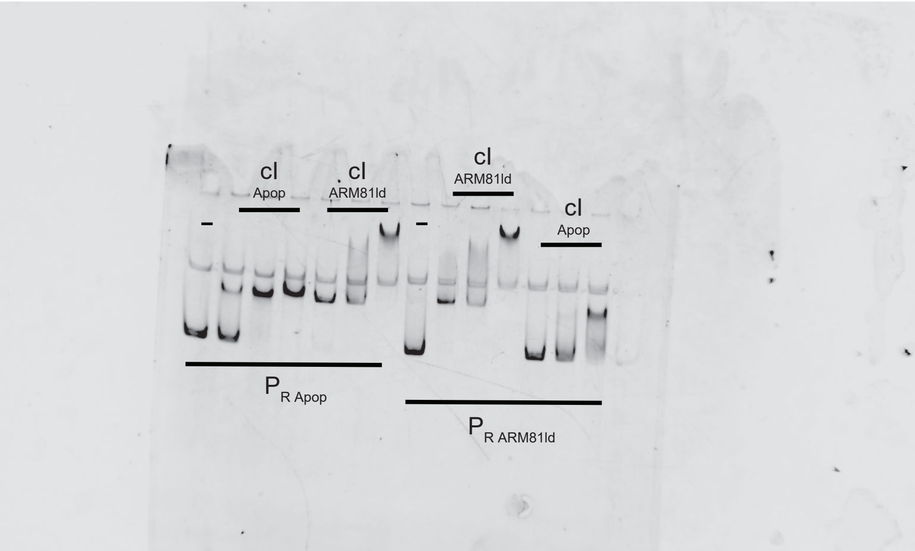

replicate 2

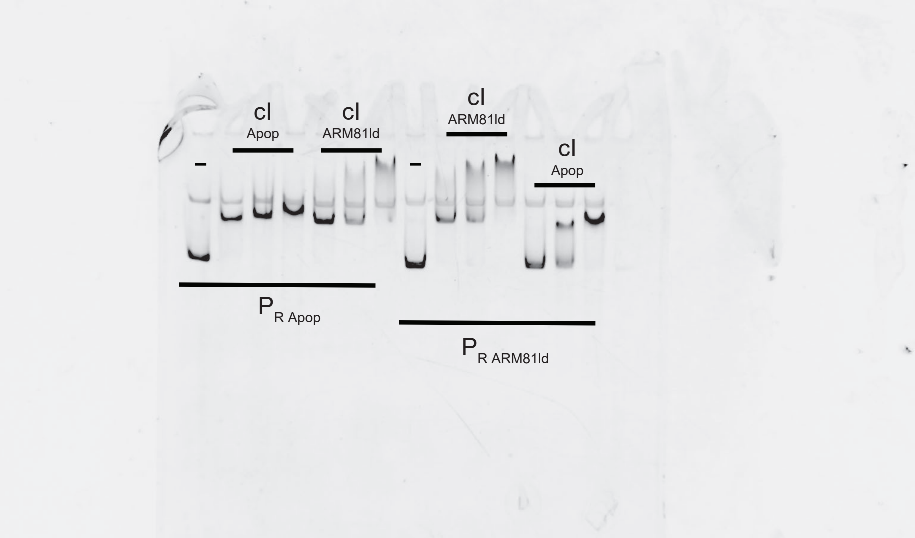

replicate 3
