# Supplementary material for: Small protein modules dictate prophage fates during polylysogeny
Source: Nature. 2023 Jul 26;620(7974):625–33. doi: 10.1038/s41586-023-06376-y (PMC10432266; doi:10.1038/s41586-023-06376-y)
Supplement: Supplementary file 2 — Reporting Summary [file 41586_2023_6376_MOESM2_ESM.pdf]

nature portfolio

Corresponding author(s): Bonnie L. Bassler

Last updated by author(s): Jun 13, 2023

Reporting Summary

Nature Portfolio wishes to improve the reproducibility of the work that we publish. This form provides structure for consistency and transparency in reporting. For further information on Nature Portfolio policies, see our Editorial Policies and the Editorial Policy Checklist.

Statistics

For all statistical analyses, confirm that the following items are present in the figure legend, table legend, main text, or Methods section.

n/a	Confirmed	
☐	☒	The exact sample size (*n*) for each experimental group/condition, given as a discrete number and unit of measurement
☐	☒	A statement on whether measurements were taken from distinct samples or whether the same sample was measured repeatedly
☒	☐	The statistical test(s) used AND whether they are one- or two-sided<br>*Only common tests should be described solely by name; describe more complex techniques in the Methods section.*
☒	☐	A description of all covariates tested
☒	☐	A description of any assumptions or corrections, such as tests of normality and adjustment for multiple comparisons
☐	☒	A full description of the statistical parameters including central tendency (e.g. means) or other basic estimates (e.g. regression coefficient) AND variation (e.g. standard deviation) or associated estimates of uncertainty (e.g. confidence intervals)
☒	☐	For null hypothesis testing, the test statistic (e.g. *F*, *t*, *r*) with confidence intervals, effect sizes, degrees of freedom and *P* value noted<br>*Give P values as exact values whenever suitable.*
☒	☐	For Bayesian analysis, information on the choice of priors and Markov chain Monte Carlo settings
☒	☐	For hierarchical and complex designs, identification of the appropriate level for tests and full reporting of outcomes
☒	☐	Estimates of effect sizes (e.g. Cohen's *d*, Pearson's *r*), indicating how they were calculated

Our web collection on statistics for biologists contains articles on many of the points above.

Software and code

Policy information about availability of computer code

Data collection	Software used to collect data generated in this study consisted of Gen5 v3.11 for collection of growth and reporter-based data; MetaGeneMark v 3.26, HMMER3 v 3.3.2, VIBRANT v1.2, MUSCLE v3.8.31, Geneious Prime v 2022.2.2, SnapGene v6, Prodigal 2.6.3, PhyML v3.3.20180621, CD-HIT v4.8.1, and BLAST 2.13.0+ for analyses of publicly available data and primer design; QuantStudio for qPCR data collection; Nikon NIS-Elements Denoise.ai for acquisition of FISH micrographs; and AlphaFold 2.1.1, PyMOL Open GL 2.1, and DALI (accessed 11/26/2022) for protein structural predictions.
Data analysis	GraphPad Prism 9 was used for analysis of growth and reporter-based experiments. FIJI v2.9.0 / 1.53r and Python v3.7.6 was used for image analyses. Custom code used to search, extract, and analyze phage genome databases based on user-defined features, and custom code for smFISH analysis are freely available and are deposited on Zenodo (doi: 10.5281/zenodo.7083051).

For manuscripts utilizing custom algorithms or software that are central to the research but not yet described in published literature, software must be made available to editors and reviewers. We strongly encourage code deposition in a community repository (e.g. GitHub). See the Nature Portfolio guidelines for submitting code & software for further information.

Data

Policy information about availability of data

All manuscripts must include a data availability statement. This statement should provide the following information, where applicable:

- Accession codes, unique identifiers, or web links for publicly available datasets
- A description of any restrictions on data availability
- For clinical datasets or third party data, please ensure that the statement adheres to our policy

Data presented in all figure panels of this study are provided in Source Data Figure files and Source Data Extended Data Figure files. Unprocessed gels and micrographs are provided in Supplementary Figure 1. All materials associated with this study are also deposited on Zenodo (doi: 10.5281/zenodo.7083051). The accession codes for proteins presented in Extended Data Figure 5b are provided in the corresponding legend and can be publicly accessed via PDB ID: 1Y7Y, 2B5A, 3G5G, and 5WOQ. Other experimental data that support the findings of this study are available without restriction by request from the corresponding author.

Research involving human participants, their data, or biological material

Policy information about studies with human participants or human data. See also policy information about sex, gender (identity/presentation), and sexual orientation and race, ethnicity and racism.

Reporting on sex and gender	Not applicable
Reporting on race, ethnicity, or other socially relevant groupings	Not applicable
Population characteristics	Not applicable
Recruitment	Not applicable
Ethics oversight	Not applicable

Note that full information on the approval of the study protocol must also be provided in the manuscript.

Field-specific reporting

Please select the one below that is the best fit for your research. If you are not sure, read the appropriate sections before making your selection.

☒ Life sciences ☐ Behavioural & social sciences ☐ Ecological, evolutionary & environmental sciences

For a reference copy of the document with all sections, see nature.com/documents/nr-reporting-summary-flat.pdf

Life sciences study design

All studies must disclose on these points even when the disclosure is negative.

Sample size	Sample size was chosen as three biological replicates, matching the standard in the microbiology field [e.g., Erez and Steinberger-Levy et al. Nature 541, 488–493 (2017)]. All datapoints displayed in this study are available in the source data for others to access and analyze. Means and standard deviations are plotted; no additional statistical analyses were performed [see D.L. Vaux "Know when your numbers are significant. Nature 492, 180–181 (2012)].
Data exclusions	No data were excluded.
Replication	At least three replicates were used for each experiment. All data points were plotted and are available in the source data file. No data were excluded, and all replicates were therefore considered "successful" measurements.
Randomization	Randomization was not formally implemented in this study, however, the choice of wells and positioning of culture tubes used in any given experiment was not pre-assigned and was therefore chosen randomly at the time of setup.
Blinding	Blinding was not formally applied in this study. The investigators setting up the assays also analyzed the data. The strains used in each experiment were assigned numbers that were cross-checked with the corresponding sample names/treatments only at the time the data were plotted.

Reporting for specific materials, systems and methods

We require information from authors about some types of materials, experimental systems and methods used in many studies. Here, indicate whether each material, system or method listed is relevant to your study. If you are not sure if a list item applies to your research, read the appropriate section before selecting a response.

Materials & experimental systems

n/a	Involved in the study	
☒	☐	Antibodies
☒	☐	Eukaryotic cell lines
☒	☐	Palaeontology and archaeology
☒	☐	Animals and other organisms
☒	☐	Clinical data
☒	☐	Dual use research of concern
☒	☐	Plants

Methods

n/a	Involved in the study	
☒	☐	ChIP-seq
☒	☐	Flow cytometry
☒	☐	MRI-based neuroimaging
